# Emerging evidence of the physiological role of hypoxia in mammary development and lactation

**DOI:** 10.1186/2049-1891-5-9

**Published:** 2014-01-21

**Authors:** Yong Shao, Feng-Qi Zhao

**Affiliations:** 1 Laboratory of Lactation and Metabolic Physiology, Department of Animal Science, University of Vermont, Burlington, Vermont 05405, USA

**Keywords:** Glucose transporter, Hypoxia, Hypoxia inducible factor, Lactation, Mammary development, Metabolism

## Abstract

Hypoxia is a physiological or pathological condition of a deficiency of oxygen supply in the body as a whole or within a tissue. During hypoxia, tissues undergo a series of physiological responses to defend themselves against a low oxygen supply, including increased angiogenesis, erythropoiesis, and glucose uptake. The effects of hypoxia are mainly mediated by hypoxia-inducible factor 1 (HIF-1), which is a heterodimeric transcription factor consisting of α and β subunits. HIF-1β is constantly expressed, whereas HIF-1α is degraded under normal oxygen conditions. Hypoxia stabilizes HIF-1α and the HIF complex, and HIF then translocates into the nucleus to initiate the expression of target genes. Hypoxia has been extensively studied for its role in promoting tumor progression, and emerging evidence also indicates that hypoxia may play important roles in physiological processes, including mammary development and lactation. The mammary gland exhibits an increasing metabolic rate from pregnancy to lactation to support mammary growth, lactogenesis, and lactation. This process requires increasing amounts of oxygen consumption and results in localized chronic hypoxia as confirmed by the binding of the hypoxia marker pimonidazole HCl in mouse mammary gland. We hypothesized that this hypoxic condition promotes mammary development and lactation, a hypothesis that is supported by the following several lines of evidence: i) Mice with an HIF-1α deletion selective for the mammary gland have impaired mammary differentiation and lipid secretion, resulting in lactation failure and striking changes in milk compositions; ii) We recently observed that hypoxia significantly induces HIF-1α-dependent glucose uptake and GLUT1 expression in mammary epithelial cells, which may be responsible for the dramatic increases in glucose uptake and GLUT1 expression in the mammary gland during the transition period from late pregnancy to early lactation; and iii) Hypoxia and HIF-1α increase the phosphorylation of signal transducers and activators of transcription 5a (STAT5a) in mammary epithelial cells, whereas STAT5 phosphorylation plays important roles in the regulation of milk protein gene expression and mammary development. Based on these observations, hypoxia effects emerge as a new frontier for studying the regulation of mammary development and lactation.

## Introduction

**Figure 1:**
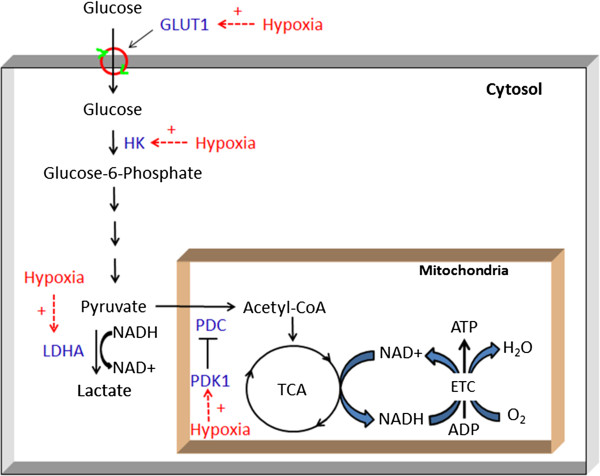
**Effects of hypoxia on glucose metabolism in cells.** Glucose, taken up by facilitative glucose transporter 1 (GLUT1), is first phosphorylated to glucose-6-phosphate by hexokinase (HK) and is then converted to pyruvate by glycolytic enzymes. Lactate dehydrogenase A (LDHA) converts pyruvate to lactate when oxygen is limited. In well-oxygenated cells, pyruvate is actively taken up by the mitochondria and converted to acetyl coenzyme A (CoA) by pyruvate dehydrogenase complex (PDC), which can be inactivated via phosphorylation by pyruvate dehydrogenase kinase 1 (PDK1). Acetyl-CoA enters the tricarboxylic acid cycle (TCA) to produce NADH, which is used to produce ATP through the electron transport chain (ETC) via the transfer of electrons to oxygen to form water. The major enzymes affected by hypoxia are indicated. “+” = stimulation.

Oxygen is critical for cellular aerobic metabolism in many higher organisms, including mammals, as it is the final electron acceptor in the electron transport chain of oxidative phosphorylation in mitochondria (Figure 1). Aerobic metabolism is 19 times more efficient in energy production than anaerobic metabolism: a molecule of glucose, the major energy source in most mammalian cells, produces up to 38 molecules of ATP in aerobic metabolism though only 2 in anaerobic metabolism [1]. In addition to efficient energy production, aerobic metabolism produces the end product H_2_O, whereas anaerobic metabolism produces lactate, which is normally removed in the liver with the requirement of oxygen in mammals. Therefore, an oxygen supply and the maintenance of oxygen homeostasis are essential in aerobic organisms.

Tissues in the body are exposed to different levels of oxygen, which are affected by the oxygen supply and tissue metabolic rate and range from near 159 mm Hg in the upper airway [the O_2_ partial pressure in the atmosphere at sea level (pO_2_); 21% of atmospheric air] to as low as 5 mm Hg (~1%) in the retina [2]. In most tissues, the oxygen tension is between 10 and 45 mm Hg (3-6%) [3,4]. Hypoxia is a deficiency of oxygen supply in the entire body (such as in high elevation exposure) or locally within a tissue when the oxygen delivery or availability is reduced due to different causes (such as tissue injury). The precise hypoxic pO_2_ is different between organs, but a venous pO_2_ of below 6% O_2_ can induce a hypoxic response in most tissues, and 0.5-1% induces the maximal effects [5].

The body and its tissues have specific mechanisms to sense oxygen levels and make the necessary adaptations for survival under hypoxic conditions. Global oxygen is sensed by central chemoreceptors located on ventrolateral surface of the medulla oblongata and peripheral chemoreceptors in the aortic and carotid bodies [6-8]. These chemoreceptors control the respiration and heart rates to adjust the oxygen supply in the entire body. In localized hypoxia and chronic hypoxia, the tissues and cells can also sense decreases in oxygen tension through transcription factor complexes known as hypoxia-inducible factors (HIFs) to restore homeostasis [2,9].

### Cellular hypoxia signaling and HIFs

**Figure 2:**
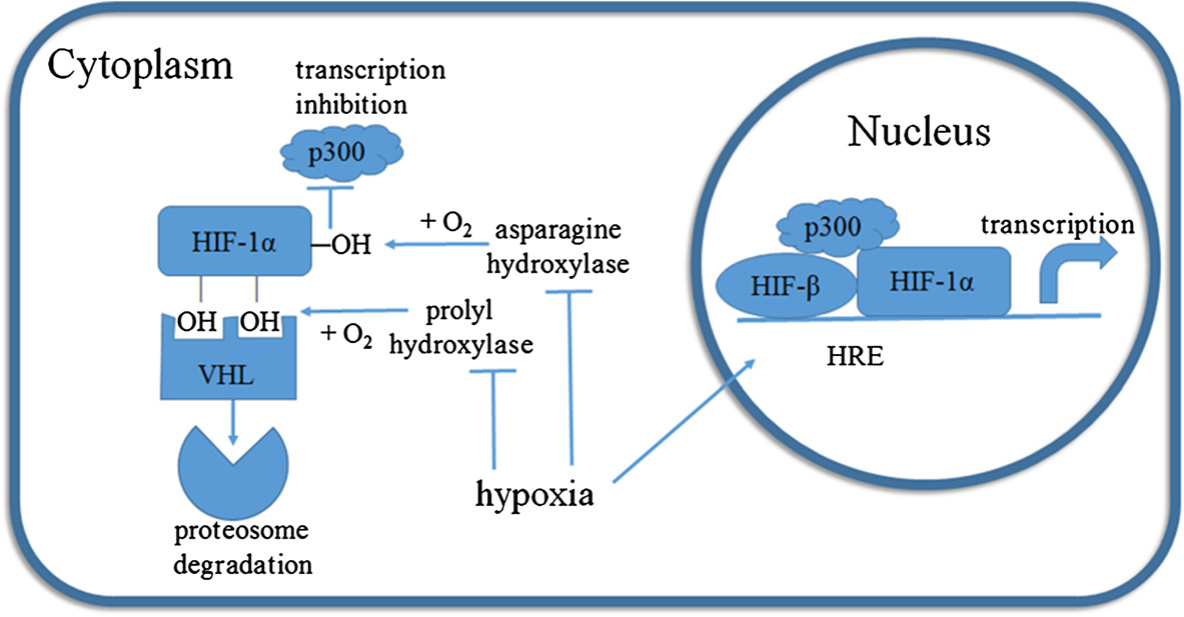
**The HIF-1α protein is stabilized under hypoxia and translocates to the nucleus to activate gene transcription.** Two proline residues of HIF-1α are hydroxylated by prolyl hydroxylase under normoxia. Hydroxylated HIF-1α is bound by the Von Hippel-Lindau (vHL) protein, leading to its ubiquitination and subsequent degradation by the proteasome. In addition, a C-terminal asparagine in the transactivation domain of HIF-1α is hydroxylated by factor inhibiting HIF-1 (FIH) under normoxia. The hydroxylation of HIF-1α blocks its interaction with the transcriptional coactivator p300 and CREB binding protein (CBP), thereby disrupting the proper assembly of HIF-1 at the hypoxia response element (HRE) of its target genes. Without oxygen, the proline and asparagine residues of HIF-1α cannot be hydroxylated; therefore, HIF-1α protein is stabilized, translocates to the nucleus, forms a heterodimer with HIF-β and activates target gene transcription.

The effects of hypoxia in many tissues and cells are primarily mediated by HIF-1 [10]. HIF-1 is a heterodimer protein consisting of an HIF-1α subunit and an HIF-1β subunit (Figure 2). Both subunits are similar in structure and contain an N-terminal basic helix-loop-helix (bHLH) domain, a Per-ARNT-Sim (PAS) domain, and a C-terminal domain [11]. The PAS domain facilitates the heterodimerization of HIF-1α and HIF-1β [12], the bHLH domain mediates the binding of HIF-1 to the consensus hypoxia response element (HRE) 5′-RCGTG-3′(R = A or G) in target gene promoters or enhancers [9], and the C-terminus of the proteins contains a transactivation domain and recruits transcriptional co-factors. HIF-1β is constitutively expressed in cells, whereas HIF-1α is under tight regulation by oxygen levels [13]. Under normoxic conditions, specific proline residues of HIF-1α are hydroxylated by prolyl hydroxylase domain proteins (PHDs) [14] (Figure 2), and hydoxylated HIF-1α is bound by the Von Hippel-Lindau (vHL) protein, a component of the E3 ubiquitin ligase complex, leading to HIF-1α ubiquitination and subsequent proteasomal degradation [15-17] (Figure 2). Thus, the transcriptional activity of HIF-1 is primarily determined by the cellular level of HIF-1α protein.

In addition to HIF-1α degradation, normoxia also regulates the transcriptional activity of HIF-1α, where a C-terminal asparagine in the transactivation domain of HIF-1α is hydroxylated by factor inhibiting HIF-1 (FIH). This hydroxylation of HIF-1α blocks its interaction with the transcriptional coactivator p300 and CREB binding protein (CBP) [18,19], thus disrupting the proper assembly if HIF-1 at the HRE.

Cells sense oxygen availability through PHDs and FIH, both of which are 2-oxoglutarate-dependent dioxygenases that require oxygen as a substrate. One oxygen atom is used to hydroxylate a proline or asparagine of HIF-1α, and the other oxygen atom is added to the co-substrate α-ketoglutarate, converting it to carbon dioxide and succinate [17,20]. Therefore, under hypoxic conditions, the activities of PHDs and FIH are repressed, and HIF-1α is stabilized and able to translocate to the nucleus, heterodimerize with HIF-1β, and activates target gene transcription.

In addition to being regulated by oxygen stress, HIF-1α has been shown to be regulated by other stimuli, including nitric oxide, reactive oxygen species, nutrient stress, and glycolytic intermediates (e.g., pyruvate and lactate) [10,21-23]. Indeed, glycolytic intermediates may act as competitors of the PHD substrate 2-oxoglutarate, preventing PHD hydroxylation of HIF-1α.

HIF isoforms in addition to HIF-1α and HIF-1β have been identified [2,24]. There are currently three structurally related HIF-α isoforms that share a similar functional mechanism [2]. In contrast to the ubiquitous expression of HIF-1α, the expression of HIF-2α is commonly found in tissues involved in oxygen delivery, such as endothelial cells and cardiac myocytes [25]. The various isoforms may also have selective transcriptional targets [2]. Additionally, HIF-3α lacks the transactivation domain and thus may play a dominant-negative role [26]. HIF-1α deletion in mice is embryonic lethal, with cardiac and vascular defects and decreased erythropoiesis [27-29]; in contrast, HIF-2α deletion is either embryonic lethal, or death occurs shortly after birth due to respiratory failure or mitochondrial dysfunction [30].

### Physiological responses of tissues to hypoxia

All nucleated cells have the ability of physiological alterations to adapt to transient or chronic hypoxia. These changes include enhancing glucose uptake, metabolic switching from oxidative phosphorylation to anaerobic glycolysis to reduce oxygen consumption, and increasing oxygen delivery by promoting angiogenesis and erythropoiesis.

### Glucose uptake

Glucose utilization in most mammalian cells is controlled by its uptake. Glucose transport across the plasma membrane of most mammalian cells is mediated by a family of facilitative glucose transporters (GLUTs), which includes 14 structurally related members designated GLUT1-12, GLUT14, and HMIT (H^+^/myo-inositol co-transporter) [31]. Each transporter has a tissue-specific distribution and distinct kinetic properties and exhibits differential regulation by ambient glucose, oxygen levels, and hormones [31]. Glucose transport is stimulated by hypoxia in many cell types, and this response is largely mediated by enhancing GLUT1 and GLUT3 transcription through HIF-1α activation [32-34]. An HRE identified in an enhancer region located approximately 2.7 kb upstream of the transcription start site in mouse and rat GLUT1 genes is reported to convey the response to hypoxia [35,36].

In addition to the direct regulation of GLUT transcription by HIF-1α, hypoxia may also stimulate glucose uptake by secondary mechanisms, such as by inhibiting oxidative phosphorylation in mitochondria. The exposure to hypoxia results in the inhibition of oxidative phosphorylation, which, without the reduction of oxygen tension, in turn stabilizes GLUT1 mRNA and up-regulates GLUT1 expression through enhancer elements that are independent of HIF binding [35,36]. A serum-responsive element (SRE) located approximately 100 nucleotides upstream of an HRE in the mouse GLUT1 gene conveys the responses to mitochondrial inhibitors [36]. In the rat GLUT1 gene, a 666-bp sequence at 6 kb upstream of the transcription start site responds to the inhibition of oxidative phosphorylation [35]. Furthermore, the induction of GLUT1 expression by low oxygen availability is rapid, whereas the half-life of GLUT1 mRNA is not affected. In contrast, the up-regulation of GLUT1 mRNA by the inhibition of oxidative phosphorylation is a delayed response, and the GLUT1 mRNA half-life increases significantly [35].

### Metabolism

In aerobic metabolism, glucose is first converted to two pyruvates through glycolysis in the cytoplasm; pyruvate dehydrogenase complex (PDC) in the mitochondria then catalyzes the conversion of pyruvate to acetyl-CoA, which enters the tricarboxylic acid (TCA) cycle and produces ATP via oxidative phosphorylation (Figure 1). However, when the availability of O_2_ becomes limited, cells switch from oxidative metabolism to anaerobic glycolysis by up-regulating the expression of several key glycolytic genes, including hexokinases 1 and 2 (HKs), lactate dehydrogenase A (LDHA), and pyruvate dehydrogenase kinase 1 (PDK1) [2,4,13]. The up-regulation of glycolytic gene expression is also mediated by HIF-1 via direct binding to promoters or enhancers [37-40].

In addition to preventing substrates from entering oxidative phosphorylation, hypoxia also regulates mitochondrial activity: it regulates cytochrome c oxidase (COX) by switching isoform COX4-1 to COX4-2, which can use the limited oxygen more efficiently [41]. Moreover, hypoxia inhibits mitochondrial biogenesis by decreasing c-Myc activity [42] and induces mitochondrial autophagy by up-regulating BNIP3 [43].

### Angiogenesis and erythropoiesis

Another strategy against hypoxia is to increase oxygen delivery, which is performed in two ways. i) Hypoxia induces angiogenesis, the growth of new blood vessels, to deliver more oxygen and nutrients to hypoxic tissues, a process that is primarily mediated via the up-regulation of vascular endothelial growth factor (VEGF) expression by HIF [44]. Hypoxia-induced angiogenesis plays important roles in wound healing, inflammation, and pregnancy. ii) In systemic hypoxia, genes related to red blood cell production and oxygen transport are up-regulated [45]. Erythropoietin (EPO) was the first gene shown to be regulated by hypoxia, and research on the underlying mechanism led to the discovery of HIF [46,47]. HIF increases the expression of EPO in the kidney and liver, stimulating the production of red blood cells in the bone marrow. Hypoxia also induces other proteins involved in iron uptake and utilization to support the production of hemoglobin [47].

In addition to the above changes, hypoxia can also induce changes in gene expression to maintain a more alkaline intracellular pH to overcome the hostile acidic extracellular environment resulted from lactate accumulation [2].

### Pathological implications of hypoxia in cancer and other diseases

Many studies on the effects of hypoxia have been conducted in cancer cells [48]. Indeed, hypoxia is a characteristic feature of malignant solid cancers, such as breast cancer. Cancer cells can grow and proliferate without external growth signals and are insensitive to growth suppressors and resistant to cell death [49]. Thus, highly aggressive and rapidly growing tumors are exposed to hypoxia as a consequence of an inadequate blood supply, which in turn plays a pivotal role in promoting tumor progression and the resistance to therapy. During tumor growth, hypoxia provides an array of effects to favor tumor survival under the hypoxic condition. These effects include gene expression changes to suppress apoptosis and support autophagy [50,51], genotype selection of cells with diminished apoptotic potential [52], increased GLUT expression and glucose uptake [53], anabolic metabolism switching [54], and the loss of genomic stability through the down-regulation of DNA repair [55] and possibly increased reactive oxygen species (ROS) production [56]. In addition, hypoxia can also suppress immune responses [57] and enhance tumor angiogenesis, vasculogenesis, the epithelial-mesenchymal transition (EMT), invasiveness, and metastasis [2,58]. These effects may also play roles in the resistance to cancer therapy [59]. Due to its multiple contributions to tumor progression, hypoxia has been suggested to be a negative prognostic factor for patient outcomes [60], and many studies have shown that endogenous hypoxia markers (e.g., HIF-1α, GLUT1, CA IX) are correlated with poorer outcomes and more aggressive malignancies [60,61]. Thus, it is not surprising that hypoxia has been a high priority target for cancer therapy [59].

In addition to cancer, hypoxia is implicated in other human diseases, such as ischemic heart disease (IHD), stroke, kidney disease, chronic lung disease, and inflammatory disorders [62]. Furthermore, cellular hypoxia may be a key factor in increasing glucose uptake by adipocytes, contributing to adipose tissue dysfunction in obesity [63,64].

### Involvement of hypoxia in physiological processes

Studies have also demonstrated that HIF-1α is required for embryonic development and the development and growth of several murine tissues, implying a role for hypoxia in these physiological processes. Most mammalian embryos develop at oxygen concentrations of 1% - 5%, thus hypoxia and HIF play important roles in embryogenesis and placenta development by regulating gene expression, cell behaviour, and cell fate [65]. HIF-1α null mice die by embryonic day (E) 10.5 due to an impaired circulatory system [13]. HIF-1α deletion in the cartilaginous growth plate of developing bone leads to death of cells in the interior of the growth plate, indicating that hypoxia plays an important role in chondrogenesis [66]. Hypoxia promotes angiogenesis and osteogenesis in bone by elevating the VEGF levels induced by HIF-1α in osteoblasts, as HIF-1α overexpression results in extremely dense, heavily vascularized long bones and a high level of VEGF, whereas the lack of HIF-1α in osteoblasts leads to significantly thinner and less vascularized long bones [67]. Hypoxia inhibits adipogenesis and the conditional knockout of HIF-1α in mouse embryonic fibroblasts impairs the hypoxia-mediated inhibition of adipogenesis [68]. In HIF-1α-deficient mice, hematopoietic stem cells (HSCs) in bone marrow lose cell cycle quiescence, and HSC numbers decrease [69]. B lymphocyte development is abnormal in HIF-1α-deficient chimeric mice, inducing autoimmunity [70].

Below, we introduce the emerging evidence of the involvement of hypoxia in mammary development and lactation.

### Mammary development and lactation

The development of the mammary gland can be divided into three stages, embryo, puberty, and pregnancy, and the major development occurs during postnatal stages. In mice, two milk lines form between the fore and hind limbs of an E 10.5 embryo, and five pairs of placodes arise along each of the two milk lines at E 11.5. The placodes then invaginate into the underlying mesenchyme to form small bulb-shaped buds. The mammary epithelial cells of the buds proliferate from E 15.5 to form sprouts that penetrate to the underlying fat pad and form the rudimentary ductal tree and teats [71,72]. Canonical Wnt signaling plays an important role during embryonic mammary development: Wnt 6, Wnt 10a, and Wnt 10b are expressed during this period, and the inhibition of Wnt signaling results in impaired placode formation [72,73]. From birth to puberty, the mammary gland first undergoes isometric growth (with the same rate as the body), followed by allometric growth (2-4X faster than body fat deposition). Robust duct branching then begins at the onset of puberty. Terminal end buds (TEBs) form from the tip of the rudimentary ductal tree of the mouse mammary gland and drive pubertal mammary development. TEBs are highly proliferative, penetrating further into the fat pad, and the bifurcations of TEBs form the primary ducts. TEBs continue to invade into the fat pad until the primary ducts reach the border of the fat pad, and the secondary ducts then branch laterally from the primary ducts until the fat pad is filled with extensive ducts [74]. In ruminants, the terminal ductal lobular units (TDLUs) are the characteristic structures of postpubertal mammary development. During this stage, estrogen and growth hormone regulate duct branching, as the knock-out of estrogen receptor (ER) α or growth hormone receptor was found to impair duct development during puberty [75,76]. During the early stage of pregnancy, short tertiary side-branches form along the ductal system developed during puberty, a process that is regulated by progesterone, progesterone receptor, and downstream Wnt 4 signaling [77,78]. The mammary epithelial cells then proliferate rapidly to form a lobuloalveolar structure within the ductal branches. Prolactin is important for alveolar morphogenesis, as the knock-out of prolactin receptor or STAT5a, a major prolactin downstream-signaling molecule, results in lobuloalveolar defects [79,80]. During late pregnancy, the mammary epithelial cells differentiate, and lactogenesis occurs under the synergistic effects of prolactin, glucocorticoids, and insulin [81]. Milk protein genes and lipogenic genes are expressed, and lipid droplets form in the epithelial cells. At the onset of lactation, the peak blood levels of lactogenic hormones and withdrawal of progesterone lead to copious milk secretion [81]. Extensive angiogenesis to supply nutrients is associated with all stages of mammary development [82]; in particular, extensive capillaries form a basket-like architecture to surround the alveoli during lobuloalveolar development. The vascular density doubles from day 18 of pregnancy to day 5 of lactation in mice [83]. In consistent with vascular development, the expression of VEGF and VEGF receptor in the rodent mammary gland increases dramatically during pregnancy and lactation and decreases during involution [84].

### Oxygen uptake in the mammary gland during mammary development and lactation

The mammary gland has high metabolic rates during development and lactation and is thus considered to be a benign, highly regulated tumor [85]. Although extensive studies have been performed in the pathology of hypoxia in breast cancer, limited attention has been given to oxygen utilization in normal mammary development and lactation, and reports on mammary oxygen uptake have been largely limited to only a few early studies [86-88]. The average O_2_ uptake of a lactating mammary gland is 0.51-0.73 μmol/min/g in goats and 2.06 in rats [86-88]. Mammary O_2_ uptake is steadily increased during late pregnancy and reaches the highest levels in early lactation [89]. The O_2_ uptake in lactating goats is twice that in the preparturient goat, and there is a correlation between mammary O_2_ uptake and milk secretion [87,88]. Starvation results in the virtual cessation of milk production, with a 75% reduction in mammary oxygen uptake [86], whereas growth hormone administration increases mammary oxygen uptake [90]. In 10-wk virgin mice, the pO_2_ level in the mammary fat pad is, on average, 13.0 mm Hg (~2%), which is considerably lower than in muscle (29.1 mm Hg, ~4%) [91]. However, a recent study using phosphonated trityl probes in mice reported an average mammary gland tissue pO_2_ of 52 mmHg [92], which is consistent with the pO_2_ value reported in normal breast tissue [93]. Nevertheless, it is likely that the mammary gland develops chronic hypoxia during the rapid mammary development in late pregnancy and in early lactation because the oxygen consumption increases in these periods to meet the increased metabolic rates.

To examine possible hypoxic conditions in the mammary gland during mammary development, we recently injected the hypoxia marker pimonidazole HCl into mice from the virgin state to the early lactation state. Pimonidazole HCl is a chemical that forms adducts with thiol groups in proteins, peptides, and amino acids in hypoxic cells (http://www.hypoxyprobe.com/), and these pimonidazole adducts can be detected with specific antibodies. Immunohistochemical staining of the mouse mammary glands from different stages showed a hypoxic mammary gland in all examined stages, but the staining was stronger during late pregnancy and early lactation (Shao and Zhao, unpublished preliminary observations), indicating a more hypoxic condition in these stages.

### Emerging evidence of a role of hypoxia in mammary development and lactation

Consistent with the hypoxic condition in the mammary gland during development and lactation, there is emerging evidence that supports possible physiological roles of hypoxia in these processes.

### 1) Selective deletion of HIF-1α in the mouse mammary gland results in impaired mammary development and lactation

The most compelling evidence of hypoxia’s role in mammary development and lactation is from a targeted gene-knockout study by Seagroves et al. (2003) [94]. Because the whole-genome knockout of the *HIF-1α* gene is embryonic lethal, Seagroves et al. specifically removed the *HIF-1α* gene from the mammary epithelial cells (MECs) using mouse mammary tumor virus (MMTV) promoter-directed cre-lox technology. No morphological defects were observed in the *HIF-1α*^-/-^ glands until day 15 of pregnancy when the mammary gland is well into secretory differentiation. By day 15 of pregnancy, striking histological differences became obvious between the wild-type and *HIF-1α*^-/-^ glands. In particular, the *HIF-1α*^-/-^ glands had smaller alveoli with no protein or lipid droplets due to impaired mammary differentiation. In addition, the expression of milk proteins β-casein and α-lactalbumin was decreased by over 50% in these glands. Surprisingly, the *HIF-1α*^-/-^ glands showed no abnormality in microvessel pattering and vascular density, despite the important role of hypoxia in angiogenesis [94]. However, this observation could be explained by *HIF-1α* only being deleted in MECs and not in other cell types, such as endothelial and stroma cells, and these cells could still respond to the hypoxia conditions and retain intact angiogenesis.

Furthermore, consistent alveolar defects and trapped large lipid droplets were observed in the *HIF-1α*^-/-^ glands during lactation [94]. The glands weighed ~50% of the wild-type glands at mid-lactation; additionally, the *HIF-1α*^-/-^ animals produced less milk than the wild-type controls, and their milk was more viscous and contained significantly elevated fat and ion contents.

These data clearly indicate that, although the expression of HIF-1α in MECs is not required for early mammary development (mainly ductal morphogenesis), it is essential for mammary secretory differentiation, milk production, and lipid secretion, implying a role for hypoxia in these physiological processes.

### 2) Hypoxia increases glucose uptake and GLUT1 expression in MECs

As in all mammalian cells, glucose is an important source of energy and NADH and also serves as a substrate for lipid, protein, and nucleotide syntheses in MECs. Glucose is also the major and an essential precursor of lactose synthesis in the lactating MECs. Mammary glucose uptake increases gradually from late pregnancy and peaks at early lactation [87,89], and mammary glucose transport activity increases approximately 40-fold from the virgin state to mid-lactation state in mice [95]. Glucose uptake in the mammary gland is mediated by facilitative glucose transporters (GLUTs) [31,96]. Mammary cells mainly express GLUT1, GLUT8, and GLUT 12, with GLUT1 being the predominant isoform [97], and there is a dramatic increase in mammary GLUT expression from late pregnancy to early lactation [97], concomitantly with mammary glucose uptake. We originally hypothesized that the lactogenic hormones (prolactin, glucocorticoids, insulin, and estrogen) are responsible for stimulating GLUT expression during lactogenesis. However, our recent study challenged this hypothesis because these hormones failed to stimulate GLUT expression in bovine mammary explants and primary MECs, even though they were able to dramatically stimulate the expression of milk protein and lipogenic genes [98].

We, thus, hypothesized that the mechanism underlying the increase in GLUT expression in MECs during the transition period from pregnancy to lactation involves hypoxia signaling through hypoxia inducible factor-1α (HIF-1α). To test this hypothesis, we recently studied the effects of hypoxia on GLUT expression in bovine MECs. Hypoxia (below 5% O_2_) significantly stimulated glucose uptake and GLUT1 mRNA and protein expression in bovine MECs yet decreased GLUT8 mRNA expression in these cells (Shao and Zhao, unpublished observations). A robust induction of HIF-1α protein was observed in the bovine MECs, consistent with the observation in mouse MECs [94]. Furthermore, an siRNA against HIF-1α completely abolished the up-regulation of GLUT1 by hypoxia but had no effect on GLUT8 expression (Shao and Zhao, unpublished observations).

Consistent with our study, the expression of GLUT1 in mouse MECs is HIF-1α-dependent in a stage-dependent manner *in vivo*. GLUT1 expression decreases by 60% in *HIF-1α*^-/-^ glands by day 16 of pregnancy, whereas no difference was observed at mid-lactation [94]. In addition, it has been shown that prolonged hypoxia stimulates GLUT1 expression in bovine endothelial cells [99,100].

Taken together, the above evidence clearly show that MECs and mammary endothelial cells are responsive to hypoxia (as high as 5% O_2_) through HIF-1α.

### 3) Possible interactions of HIF-1α with other signaling pathways in the mammary gland

The signal transducer and activator of transcription 5 (STAT5) is essential for mammary gland differentiation and lactation [101,102] and is mainly activated by prolactin and its receptor in the mammary gland. After binding prolactin, the prolactin receptor phosphorylates and activates Janus kinase (JAK) 2. JAK2 then phosphorylates STAT5, and STAT5 dimerizes and translocates to the nucleus to stimulate target genes’ expression [103,104]. In addition to the prolactin pathway, STAT5 can also be activated by epidermal growth factor (EGF), growth hormone, insulin growth factor (IGF), estrogen, and progesterone signaling pathways in the mammary gland [105]. STAT5 controls the population of luminal progenitor cells that will differentiate to alveolar cells [106,107]. During lactogenesis and lactation, the prolactin-STAT5 pathway controls the expression of milk protein genes and lipogenic genes [81,108]. STAT5-null mice have impaired mammary alveologenesis due to a reduction in the mammary luminal progenitor cell population and exhibit impaired milk protein gene expression [109]. It has been reported that hypoxia and HIF-1α can induce STAT5 phosphorylation and enhance its DNA-binding activity in mammary epithelial cells and breast cancer cells [110,111]. Thus, hypoxia may be involved in mammary development and lactation by regulating STAT5 activity.

Notch signaling responds to extrinsic or intrinsic developmental cues and regulates multiple cellular processes, such as stem cell maintenance, cell fate specification, and differentiation [112]. Upon ligand binding, the Notch protein is cleaved by presenilin/γ-secretase to release the active intracellular domain (ICD), which translocates to the nucleus to regulate the transcription of target genes [113]. In the mammary gland, Notch represses mammary stem cells expansion in the basal cell compartment [114] and promotes luminal cell fate specification and prevents myoepithelial cell lineage during pregnancy [114,115]. Hypoxia prevents differentiation in various stem and precursor cells, and research has shown that HIF-1α interacts with the Notch ICD to activate Notch target genes, inhibiting the differentiation of myogenic and neutral precursor cells [116]. In addition, FIH negatively regulates Notch by hydroxylating two asparagine residues of ICD [117]. Therefore, hypoxia and Notch signaling may cross-talk to regulate cell differentiation in the mammary gland.

Wnt proteins are involved in multiple events during embryogenesis and adult tissue development, with effects on cell fate specification, differentiation, mitogenic stimulation, and stem cell self-renewal [118]. The Wnt signaling pathways include the canonical pathway involving β-catenin and noncanonical pathways; the canonical pathway has been investigated intensively and best characterized. Wnt proteins bind to the cell surface receptor Frizzled in conjunction with low-density lipoprotein receptor-related proteins (LRPs), which transduce the signal to intracellular proteins, leading to the stabilization of β-catenin. β-Catenin then translocates to the nucleus and interacts with transcription factor lymphoid enhancer-binding factor 1/T cell-specific transcription factor (LEF/TCF) to affect target gene transcription [118]. Wnt signaling is essential for mammary rudiment formation in embryos, as the lack of TCF or the overexpression of the Wnt inhibitor Dkk-1 impairs the formation of mammary placodes [73,119,120]. Wnt signals drive ductal branching during pubertal mammary development, because the deletion of LRP reduces duct branching and the overexpression of LRP increases duct branching in the virgin mouse mammary gland [121-123]. Wnt-4 works as a paracrine factor downstream of progesterone signaling during pregnancy to stimulate lobular alveolar development [77,124]. During mammary development, both ductal and alveolar epithelial cells originate from mammary stem cells, and Wnt signaling serves as a rate-limiting self-renewal signal to maintain mammary stem cells [125]. Most stem cells reside in hypoxic niches, and research has shown that Wnt signaling is modulated by hypoxia in embryonic stem cells and neural stem cells by enhancing β-catenin activation and increasing LEF/TCF proteins [126]. Such interactions may also exist in the mammary gland.

## Conclusion

**Figure 3:**
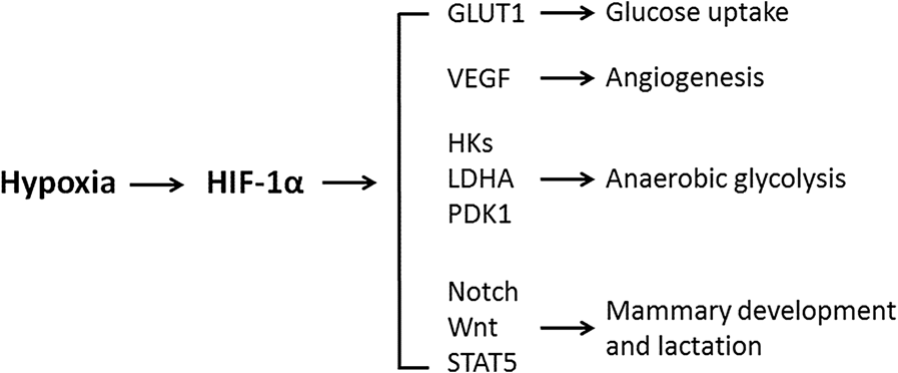
**Schematic diagram of proposed effects of hypoxia on the mammary gland.** Hypoxia stabilizes the hypoxia-inducible factor-1α (HIF-1α), which stimulates: i) glucose uptake through enhancing the expression of facilitative glucose transporter 1 (GLUT1), ii) angiogenesis through enhancing the expression of vascular endothelial growth factor (VEGF), iii) anaerobic glycolysis through up-regulating the expression of several key glycolytic genes, including hexokinases 1 and 2 (HKs), lactate dehydrogenase A (LDHA), and pyruvate dehydrogenase kinase 1 (PDK1), and iv) mammary development, differentiation and lactation through regulation of Notch, Wnt and STAT5 signaling pathways.

Increased oxygen and energy requirements, nutrient stress, extensive angiogenesis, and metabolic switching to glycolysis [127] in the mammary gland during the transition period from pregnancy to lactation make hypoxia a possible regulator of these processes. The possible effects of hypoxia on the mammary gland include, but not limit to the stimulations of: i) glucose uptake through enhancing the expression of GLUT1, ii) angiogenesis through enhancing the expression of VEGF, iii) anaerobic glycolysis through up-regulating the expression of several key glycolytic genes, including HKs, LDHA, and PDK1, and iv) mammary development, differentiation and lactation through regulation of Notch, Wnt and STAT5 signaling pathways (Figure 3).

However, based on above evidence, it is important to note that, although HIF-1α is clearly required in normal mammary development and lactation, there is a possibility that hypoxia per se may not be the major or only stimulus of HIF-1α activity in the mammary gland. As mentioned above, in addition to being regulated by hypoxia, HIF-1α stability has been shown to be regulated by other stimuli, including nitric oxide, reactive oxygen species, nutrient stress, and glycolytic intermediates [10,21-23]. Furthermore, several studies have shown that hypoxia causes mammary epithelial disorganization and induces a cancer cell-like phenotype in human MECs [128-130]. Thus, to link an effect of hypoxia to mammary development and lactation, it is essential to quantitatively measure oxygen tension changes in the mammary gland from pregnancy to lactation. Our hypothesis is that a small degree of the hypoxic condition is involved in mammary development and lactation from late pregnancy to early lactation, whereas more severe hypoxia is involved in breast cancer development.

## Abbreviations

FIH: Factor inhibiting HIF-1; GLUT: Facilitative glucose transporter; HIF: Hypoxia-inducible factor; HRE: Hypoxia response element; LDHA: Lactate dehydrogenase A; MECs: Mammary epithelial cells; PDC: Pyruvate dehydrogenase complex; PDK1: Pyruvate dehydrogenase kinases 1; PHD: Prolyl hydroxylase domain protein; SRE: Serum responsive element; STAT5: Signal transducer and activator of transcription 5; TCA: Tricarboxylic acid cycle.

## Competing interests

The authors declare that they have no competing interests.

## Authors’ contributions

YS and FQZ drafted the manuscript, and FQZ revised the manuscript. Both authors read and approved the final manuscript.
